# Estimating Everyday Neuropsychological Functioning in Multiple Sclerosis: Reliability and Validity of the Greek Multiple Sclerosis Neuropsychological Questionnaire

**DOI:** 10.1155/2018/6301535

**Published:** 2018-09-25

**Authors:** Eleni Konstantinopoulou, Panagiotis Ioannidis, Christos Bakirtzis, Virginia Giantzi, Theodora Afrantou, Dimitrios Parissis, Lambros Messinis, Grigorios Nasios, Nikolaos Grigoriadis

**Affiliations:** ^1^2nd Department of Neurology, Aristotle University of Thessaloniki, Thessaloniki 54636, Greece; ^2^2nd Department of Neurology, AHEPA University Hospital, Thessaloniki 54636, Greece; ^3^Neuropsychology Section, Department of Neurology, University of Patras, Medical School, 26504 Patras, Greece; ^4^Higher Educational Institute of Epirus, Ioannina, Department of Speech and Language Therapy, Greece

## Abstract

The Multiple Sclerosis Neuropsychological Questionnaire is a brief screening questionnaire for the assessment of everyday neuropsychological competence of patients with Multiple Sclerosis. The aim of the present study was to examine psychometric properties of the Greek version of the instrument. One hundred and three MS patients and 60 informants participated in the present study and completed the questionnaire. From the initial patient sample, 51 participants completed broadly used neuropsychological tests and measures estimating cognitive failures and depression. Moreover, after a six-month interval the MSNQ was administered to 58 patients from the initial sample in order to explore test-retest reliability. Cronbach's *α* was 0.92 and 0.93 for patient and informant forms, respectively. The patient form was correlated significantly with measures of cognitive failures and depression. Low correlations were found between the informant form and performance on cognitive tests. In regard to the patient form, significant correlation was observed between repeated administrations and, psychometrically, the three-factor structure was preferable than the one-factor structure. The present study confirms the already established pattern of correlations among the two MSNQ forms, neuropsychological test performance and depression measurements. Additional research is needed in order to define a cut-off score for the MSNQ-I providing further information about the diagnostic interpretability of the instrument.

## 1. Introduction

Cognitive impairment is frequent in MS occurring in about 45% of patients, although according to various studies there is a range in prevalence rates (40-65%) [1]. The heterogeneity of neuropsychological dysfunction among MS patients is highlighted and impairment can be observed in any disease stage affecting a range of cognitive functions such as memory, executive functions, attention, and processing speed [2].

Brief batteries of neuropsychological tests have been proposed for the assessment of cognitive functioning in MS [2]. The Multiple Sclerosis Neuropsychological Questionnaire (MSNQ) is a 15-item questionnaire for the identification of patients with possible neuropsychological impairment [3]. Apart from the self-report form (MSNQ-P), an informant form is also available (MSNQ-I). The patient-informant rating discrepancy offers information about possible impairment in self-awareness and is also associated with neuropsychiatric features [4]. Moreover, since lack of awareness of cognitive deficits is an important concern in MS, informant ratings regarding patients' neuropsychological status are valuable and should be included in standard assessment procedures [5, 6]. Moreover, previous research supports the relationship between the MSNQ-P score and depressive symptoms and suggests the implementation of the MSNQ-I for the evaluation of cognitive deficits [3, 7].

Prevalence and incidence rates of MS have been increased in Greece [8] but neuropsychological assessment is not included in routine clinical evaluations. Furthermore, screening instruments for the detection of Greek MS patients with cognitive impairment are lacking. Consequently, the aim of the present study was to examine basic psychometric properties of the Greek version of the MSNQ.

## 2. Method

### 2.1. Participants

One hundred and three patients (65 women, 63%) attending the MS center at 2nd Neurology Department, AHEPA University Hospital, Aristotle University of Thessaloniki, participated in the present study and completed the Multiple Sclerosis Neuropsychological Questionnaire (MSNQ-P). Patients were included in the study regardless of any Disease Modifying Treatment and were diagnosed by standard criteria [9]. The majority of patients (n= 84, 81%) were diagnosed with Relapsing Remitting MS (RRMS), 14 with Secondary Progressive MS (SPMS), and five with Primary Progressive MS (PPMS). Exclusion criteria were (1) current or past neurological disorder other than MS; (2) relapse within eight weeks of assessment; (3) history of, or current, psychiatric disorder; (4) history of, or current, substance abuse. Mean Expanded Disability Status Scale (EDSS) score was obtained retrospectively (within six months) from medical records and was unavailable for one patient. More than half of MS participants (n=58, 56%) completed the MSNQ-P for a second time after a six-month interval (Subgroup 1). Similarly, the majority of patients (n= 48, 82%) in this subgroup were diagnosed with RRMS, eight with SPMS and two with PPMS.

Based on both criteria of being a spouse or a parent and having daily contact with the patient, 60 informants completed the MSNQ-I (Subgroup 2). The majority of patients whose informants completed the MSNQ-I (n= 47.78%) were diagnosed with RRMS, nine with SPMS and four with PPMS.

Moreover, from the initial MS sample 51 patients (49%) completed broadly used neuropsychological tests estimating verbal memory, processing speed, and executive functions (Subgroup 3), as well as the Cognitive Failures Questionnaire (CFQ) and the Beck Depression Inventory (BDI). Again, the majority of patients (n= 44, 86%) were diagnosed with RRMS, five with SPMS and two with PPMS.  Table 1 presents demographic and clinical information for each MS subgroup, as well as for the total group of patients. Mean ratings on the MSNQ-P, the CFQ, the BDI, and mean performance on neuropsychological tests are presented in Table 2.

### 2.2. Measures

#### 2.2.1. The Multiple Sclerosis Neuropsychological Questionnaire (MSNQ)

The MSNQ is a disease specific questionnaire estimating patient's and informant's perspective of patient's everyday neuropsychological competence [2]. Both forms include 15 questions, which came up from a larger pool of items addressing the spectrum of neuropsychological symptoms occurring in MS [7]. Scoring is based on a 5-point Likert scale, ranging from 0 (does not occur) to 4 (very often, very disruptive).

#### 2.2.2. The Cognitive Failures Questionnaire (CFQ) and The Beck Depression Inventory (BDI-I)

The CFQ was developed to assess cognitive failures and errors in everyday tasks. It is a self-report questionnaire including 25 items and its scoring is based on 5-point Likert scale [10]. The BDI-I contains 21 items which estimate depressive symptoms [2].

#### 2.2.3. Neuropsychological Tests

In order to estimate objective neuropsychological performance, a brief battery of neuropsychological tests was used. Verbal memory was estimated with the Greek Verbal Learning Test (GVLT) [11] and delayed free recall score was utilized for analyses. Processing speed was assessed with the Stroop Color-Word Test (SCWT) [12–14] as well as with the Experimental Processing Speed Test (EPST), an experimental measure which was developed for the present study according to the oral version of Symbol Digit Modalities Test [15]. The total score on the first (number of correct words read in 45 seconds) and second (number of correct colors named in 45 seconds) conditions of the SCWT and the total score on EPST (number of correct answers in 90 seconds) were used for analyses. Executive functions were estimated via the interference condition of SCWT (number of correct answers in 45 seconds) and the Verbal Fluency Test (VFT, subscores on semantic and phonemic conditions) [16]. Moreover, patients completed the Digit Span test [17] and total score, as well as subscores on forward and backward conditions, was used for analyses.

### 2.3. Procedures

The Greek version of the MSNQ-P was provided by its developer (R. H. Benedict). Minor grammatical adjustments were made, in order to develop the informant form (MSNQ-I).

Patients were asked to complete the MSNQ-P and available informants completed the MSNQ-I. Both forms were administered to participants by a staff neurologist or a psychologist. Next, all patients were informed for their further potential participation requesting the administration of neuropsychological tests. At this phase of the study the presence of an appropriate informant was an inclusion criterion for participation.

Cognitive impairment was evaluated according to published Greek normative data. In order to define impairment the criterion of two standard deviations below mean was used and three cognitive measures were assumed: memory (i.e., abnormal score on CVLT), processing speed (i.e., abnormal score on color and/or word conditions of the SCWT), and executive functioning (i.e., abnormal score on the interference condition of the SCWT, on phonemic and/or semantic conditions of the VFT).

All participants gave their informed consent to participate and the study was conducted according to the ethical standards set forth in the Declaration of Helsinki.

### 2.4. Data Analysis

Spearman rank correlation coefficients were calculated in order to explore possible correlations between variables, to estimate test-retest reliability and criterion-related validity. Mann–Whitney* U* Test for independent samples and Wilcoxon signed-rank test for paired data were conducted in order to examine group differences and difference between MSNQ-P test and retest scores, respectively. Internal Consistency was estimated for both MSNQ forms via Cronbach's alpha coefficient.

Exploratory factor analysis (EFA) with direct oblimin rotation was used to investigate the factor structure of MSNQ-P. Because of the small sample of available informants, factor structure of the MSNQ-I was not explored in the present study. Eigenvalue (number of factors with eigenvalue >1) and scree plot criteria (number of factors before the break) were used to determine the number of factors. Subsequently, factor solution extracted by the EFA was further analyzed using Confirmatory Factor Analysis (CFA). The following goodness-of-fit indices were used for model selection: Chi-square (*χ*^2^), comparative fit index (CFI), Tucker-Lewis index (TLI), root-mean-square error of approximation (RMSEA), standardized root-mean-square residual (SRMR), and Akaike's information criterion (AIC). Parameters were obtained by maximum likelihood estimation (MLE). The recommended criteria for good fit are CFI and TLI close to or higher than 0.95, SRMR lower than 0.08, RMSEA lower than 0.06 (lower and upper limits of the confidence interval lower than .05 and .10, respectively), and minimum AIC [18]. According to Hu and Bentler [2], for model fit evaluation in small sample sizes (<250), the combination of CFI and SRMR fit indices is preferable to the combination of TLI and RMSEA.

Statistical analyses were performed using the Statistical Package for Social Sciences (SPSS, version 22). CFAs were conducted using the R software (version 3.2.3 for Windows). Level of significance was set at 0.05.

## 3. Results and Discussion

### 3.1. Descriptive Information and Group Differences

Clinical variables, namely, duration of disease and EDSS score, were not correlated with any MSNQ form. The two MSNQ forms were significantly correlated with each other (*r*_*s*_=.530,* p*=.000). MSNQ-P score was higher than MSNQ-I score but this difference was not significant.

Thirteen patients (25%) were impaired in one cognitive domain and two patients (3%) in two cognitive domains. Impaired performance on three cognitive domains was not observed in any patient.

### 3.2. Reliability

Cronbach's *α* was 0.92 and 0.93 for the patient and the informant MSNQ form, respectively, suggesting excellent internal consistency.

Also, test-retest reliability was high. In specific, significant correlation was observed between MSNQ-P repeated administrations (*r*_*s*_=781,* p*=.000). MSNQ-P scores were lower at retesting (after the six-month interval), but this decrease was not significant.

### 3.3. Criterion-Related Validity

The MSNQ-P was correlated with the CFQ (*r*_*s*_=.762,* p*=.000) and the BDI (*r*_*s*_=.315,* p*=.025). However, nonsignificant correlations were observed between the MSNQ-P and neuropsychological test performance.

On the contrary, the MSNQ-I was correlated significantly with performance on the Verbal Fluency, the Greek Verbal Learning, and the Digit Span tests. Nevertheless, the observed correlations were low (Table 3). The correlation between MSNQ-I and BDI scores did not reach significance (*p*>.05) and a low, yet significant, correlation was observed between the MSNQ-I and the CFQ (*r*_*s*_=. 355,* p*=.020).

### 3.4. Factor Structure

Exploratory Factor Analysis (EFA) with direct oblimin rotation was conducted in order to explore the factor structure of the MSNQ-P. Three factors were observed explaining the 65% of the total variance. According to the unrotated factor solution all items loaded onto the first factor explaining the 49% of the total variance. The total amount of variance and the amount of variance explained by each factor were not increased after rotation. However, more interpretable factor solution was observed. Items corresponding to the ability to remember of performing planned actions in the future loaded onto the first latent factor. Items related to memory and attention, as well as to uncaused emotional reactions, loaded onto the second factor. Finally, executive and problem solving items loaded onto the third factor. Four items loaded also onto a second latent factor, but observed loadings were low (<.40). Factor loadings after rotation, eigenvalues, and the amount of the variance explained by each factor are shown in Table 4.

The three-factor structure identified by the EFA was further explored using Confirmatory Factor Analysis (CFA). Moreover, an alternative model was tested, namely, the theoretically driven one-factor model [19]. The value of* χ*^2^ in both models was not significant (*p*>.05). Apart from the RMSEA fit index, the remaining fit indices were adequate in the three-factor model only (Table 5).

## 4. Discussion

According to the results of the present study both forms of the Greek MSNQ have excellent internal consistency. Moreover the MSNQ-P has high test-retest reliability. However, a nonsignificant decline was observed in MSNQ-P scores after the six-month interval. In a previous study researchers noticed a significant decline in repeated administrations which was attributed to a number of factors, such as response shift and increased awareness [20]. It should be emphasized that, within this six-month interval, which is longer than the conventional retesting interval, disease course and disease characteristics were stable (i.e., no relapses, no medication/treatment modifications).

Also inconsistent with previous findings [19], the results of EFAs and CFAs support the multidimensionality of the MSNQ-P. Nevertheless, the MSNQ-P can also be assumed as a one-dimensional questionnaire, since according to the unrotated factor solution of the EFA all items loaded onto the first factor explaining a respectable amount of the total variance. The results from the CFAs though suggest that the three-factor model fitted the data more satisfactorily. This is a reasonable evidence since the MSNQ includes items that are related to different conceptual dimensions (attention/processing speed, memory, other cognitive ability, personality, and behavior) [7].

In agreement with previous findings the MSNQ-P was highly correlated with the CFQ. Moreover, the MSNQ-P was correlated positively with the BDI, a finding which can be added on evidence underlying the susceptibility of the patient form to symptoms associated with depression [2]. It is important to highlight though that patients with a diagnosis of depressive disorder and any other psychiatric comorbidity were not participated in the present study. In regard to the MSNQ-I, low correlations were observed with neuropsychological measures of executive functioning and memory that are usually impaired in MS patients. Despite the frequency of information processing speed impairment in MS [21], in the present study “pure” measures of information processing speed were not correlated with the MSNQ-I. Nevertheless, correlations were observed between MSNQ-I score and performance on the Verbal Fluency Test which is also a time-dependent task. The lack of significant correlations between the MSNQ-I and performance on tests of processing speed could be attributed to the administration of an experimental test instead of the original SDMT. However, it should be highlighted that the MSNQ includes only one item directly focusing on the domain of processing speed.

Decreased awareness of cognitive deficits is an important concern in MS [5]. Moreover, literature suggests that self-reports of brain-damaged patients tend to underestimate cognitive impairment [22]. Thus, informant ratings regarding patient's neuropsychological status are helpful and should be included in standard assessment procedures in MS, similarly to other groups of brain-damaged patients [6]. In addition, in MS as well as in other clinical groups subjective cognitive complaints are linked to depression [3, 23, 24]. Considering the frequency of depressive symptoms in MS, the significant impact of depression on self-reports, and the susceptibility of the MSNQ-P to depression, increased scores should be treated with caution and additional information from an informant is valuable.

In spite of psychometric adequacy regarding internal consistency, test-retest reliability, construct, and concurrent validity of the Greek version of the MSNQ cut-off scores were not defined in the present study. This inadequacy is associated with the major limitation of the study concerning the small number of MS participants with severe cognitive impairment and/or the lack of a control group. Therefore, additional research is needed in order to examine the diagnostic interpretability of the instrument. However, it should be mentioned that cut-off scores for the MSNQ-P have not been defined in previous studies where low sensitivity has been repeatedly observed.

## 5. Conclusions

Cognitive impairment not only affects everyday functioning in MS [25] but also has important implications in clinical management of patients. Reliable neuropsychological screening offers essential information about neurobehavioral symptoms of the disease. However, the Greek MSNQ is not appropriate to serve as a screening tool per se, since correlations with neuropsychological test performance are low. The MSNQ scales can be used as an index of agreement between patient's and informant's perspective in order to provide important information related to awareness and to facilitate treatment and rehabilitation. Furthermore, concurrent implementation of the MSNQ with a brief and well-established measure of processing speed (i.e., the SDMT) [26] would be more appropriate for efficient cognitive screening in clinical and research settings.

## Figures and Tables

**Table 1:** Demographic characteristics and clinical data for MS subgroups and total MS group.

MS Group	n	Females	Age	Education	EDSS	Duration
			Mean (S.D)	Mean (S.D)	Median (min - max)	Mean (S.D.)
Subgroup 1	58	65.52%	40.72 (6.53)	13.10 (2.77)	4.4 (1.0-7.0)	10.65 (6.12)
Subgroup 2	60	62.19%	41.57 (7.21)	12.96 (2.54)	4.5 (1.0-7.0)	11.37 (8.03)
Subgroup 3	51	60.78%	41.36 (7.34)	13.04 (2.28)	3.0 (1.0-7.0)	10.85 (6.94)
Total	103	63.11%	42.04 (9.88)	13.22 (2.89)	4.5 (1.0-7.0)	11.21 (7.02)

**Table 2:** Descriptive information for MS participants.

	n	Mean (S.D.)
MSNQ-P	103	16.65 (12.02)
MSNQ-P *retest*	58	14.26 (10.36)
MSNQ-I	60	13.92 (11.29)
CFQ	51	12.30 (6.72)
BDI	51	10.78 (6.73)
GVLT*del*	51	11.96 (2.57)
EPST	51	45.44 (11.69)
SCWT	51	
*Word *		88.00 (15.44)
*Color *		65.92 (13.15)
*Color-Word*		40.56 (10.17)
VFT	51	
*Semantic*		53.24 (9.07)
*Phonemic*		28.57 (8.97)
DS	51	
*Total*		12.69 (3.08)
*Forwards*		6.90 (1.66)
*Backwards*		5.78 (1.99)

EDSS=Expanded Disability Status Scale, MSNQ-P=MSNQ patient form, MSNQ-I=MSNQ informant form, CFQ=Cognitive Failures Questionnaire, BDI=Beck Depression Inventory, GVLT*del*=Greek Verbal Learning Test delayed recall trial, SCWT=Stroop Color-Word Test, EPST=Experimental Processing Speed Test, VFT=Verbal Fluency Test, and DS=Digit Span.

**Table 3:** Correlation coefficients between the MSNQ and neuropsychological test performance.

	GVLT *Del*	STR *W*	STR *C*	STR *CW*	EPST	VFTS	VFTP	DS	DSF	DSB
MSNQ-I	-.303*∗*	-.050	-.175	.070	-.049	-.287*∗*	-.301*∗*	-.388*∗∗*	-.227	-362*∗∗*
MSNQ-P	-.007	-.112	-.036	.151	-.233	-.163	-.170	-.173	-.158	-.108

*∗*Correlation is significant at 0.05.

*∗∗*Correlation is significant at 0.01.

GVLT*Del*=Greek Verbal Learning Test delayed recall score, STR*W*=Stroop Color-Word Test total score on word condition, STR*C*=Stroop Color-Word Test total score on color condition, STR*CW*= Stroop Color-Word Test score on color-word condition, EPST=Experimental Processing Speed Test, VFTS = Verbal Fluency Semantic condition, VFTP=Verbal fluency phonemic condition, DS=Digit Span test Total, DSF= Digit Span Test Forwards, and DSB=Digit Span Test backwards.

**Table 4:** Rotated solution and factor loadings of MSNQ-P items.

Item no. and description	Factors		
	1	2	3
(9) Forgetting future errands	.882		
(8) Needing frequent reminders	.808		
(4) Forgetting appointments	.771		
(1) Distractibility		.863	
(5) Forgetting what is read		.768	
(14) Without cause laughing/crying		.694	
(2) Problems with listening to others	.399	.639	
(12) Failing to follow conversations	.331	.448	
(7) Forgetting instructions	.362	.432	
(6) Forgetting shows/programs		.414	.375
(15) Excessive egocentric speech			.728
(10) Coherent question answering			.671
(11) Failing to track two tasks at once			.650
(13) Impulse control			.549
(3) Slowed problem processing			.460

Eigenvalues	7.453	1.218	1.101
% of variance	49.69	8.12	7.34
Cronbach's *α*	.874	.889	.786

**Table 5:** Goodness of-fit indices of CFA models.

Model	*χ* ^*2*^	df	CFI	TLI	RMSEA	RMSEA 90% CI	SRMR	AIC	BIC
One-Factor	205.17	90	.85	.83	.11	.091 - .132	.07	4136	4130
Three-Factor	135.61	87	.94	.93	.07	.048 - .097	.05	4073	4055

## Data Availability

The data used to support the findings of this study are available from the corresponding author upon request.
